# Recent applications of chiral calixarenes in asymmetric catalysis

**DOI:** 10.3762/bjoc.14.117

**Published:** 2018-06-08

**Authors:** Mustafa Durmaz, Erkan Halay, Selahattin Bozkurt

**Affiliations:** 1 Department of Chemistry Education, Ahmet Kelesoglu Education Faculty, Necmettin Erbakan University, 42090 Konya, Turkey; 2 Department of Chemistry and Chemical Processing Technologies, Banaz Vocational School, Usak University, Usak, Turkey; 3 Scientific Analysis Technological Application and Research Center (UBATAM), Usak University, Usak, Turkey; 4 Vocational School of Health Services, Usak University, 64200 Usak, Turkey

**Keywords:** asymmetric catalysis, chiral calixarene, organocatalyst, supramolecular catalyst

## Abstract

The use of calixarenes in asymmetric catalysis is receiving increasing attention due to their tunable three-dimensional molecular platforms along with their easy syntheses and versatile modification at the upper and lower rims. This review summarizes the recent progress of synthesis and use of chiral calixarenes in asymmetric syntheses which emerged later than 2010.

## Introduction

The catalysis of organic reactions by macrocyclic host compounds is a longstanding proposed application of supramolecular chemistry and utilizes the use of noncovalent interactions in catalytic systems to achieve higher reaction rates, more selective catalysts, or a larger numbers of ligands [1]. The formation of noncovalent interactions due to the selective binding of the substrate by the catalyst results in conversion of the reactants into the products and causes an activity and selectivity increase. Especially the use of supramolecules in asymmetric catalysis has received considerable interest and witnessed significant progress in recent years [2–4].

Calixarenes are considered as the third generation of supramolecular hosts after cyclodextrins and crown ethers [5–6]. Due to their easy preparation and readily modification at either the upper and/or lower rims of the molecular skeleton, they have been widely used for construction of artificial host molecules and found applications in various fields like molecular recognition, sensing, self-assembly, catalysis, nanoscience, drug delivery and separation science [7–21].

In general, chirality can be introduced into the calixarene platform either by incorporation of a chiral group or by asymmetric placement of achiral substituents, creating chirality associated with form. This is termed inherent chirality [22]. Although chiral calixarenes prepared by these ways have been widely used in chiral recognition [23–24], the use of calixarenes in asymmetric catalysis is a new emerging area.

This review attempts to illustrate a systematic overview of recent progress in asymmetric catalysis using different chiral calixarene derivatives. Since several reviews on this topic have been published [25–31], the main focus will be on recent progress from 2010 through to the beginning of 2018. The reports on the synthesis and catalytic applications of calixarenes have been classified according to the individual organic reaction in the following order: phase-transfer catalysis, Henry reaction, Suzuki–Miyaura cross-coupling and Tsuji–Trost allylic substitution, hydrogenation, Michael addition, aldol and multicomponent Biginelli reactions, epoxidation, Meerwein−Ponndorf−Verley reduction, aza-Diels−Alder and epoxide ring-opening reaction.

## Review

### Phase-transfer catalysis

**Figure 1:**
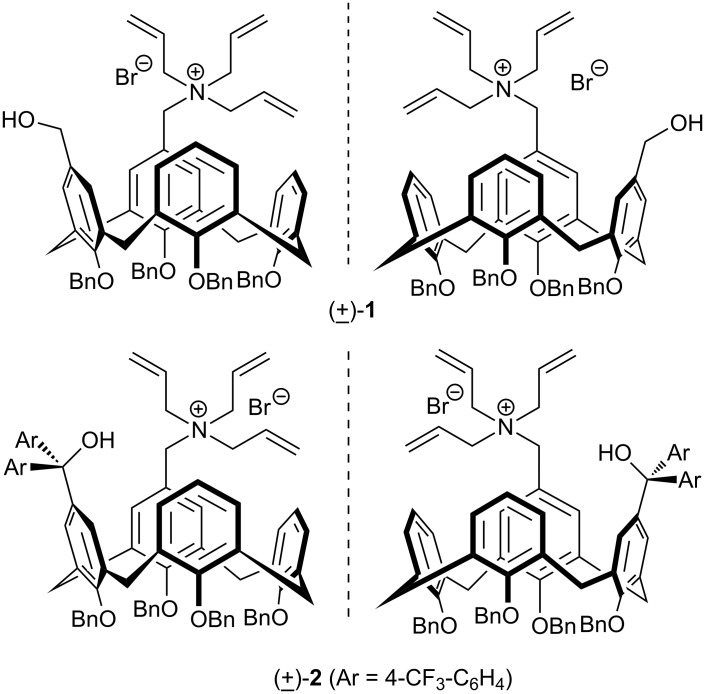
Inherently chiral calix[4]arene-based phase-transfer catalysts.

For the past three decades, asymmetric phase-transfer catalysis utilizing chiral quaternary ammonium salts has attracted great interest as a synthetic strategy since it provides quick access to a large number of enantiopure compounds employing only catalytic amounts of chiral phase-transfer agents [32–34]. Although the literature contains examples of calixarene derivatives used as phase-transfer catalysts (PTCs) [35], the first asymmetric quaternary ammonium salts derived from cinchona alkaloids based on the calixarene skeleton were prepared by our group. These were applied to the asymmetric alkylation reaction of benzophenone imine of glycine ester which has become one of the benchmark reactions for examining the performance of new phase-transfer catalysts [36]. Later in 2010, Shirakawa and Shimizu reported the synthesis of novel inherently chiral calix[4]arenes (±)-**1** and (±)-**2** containing a quaternary ammonium moiety together with a hydroxymethyl and diarylmethanol moiety in an optically pure form, respectively (Figure 1) [37].

**Scheme 1:**
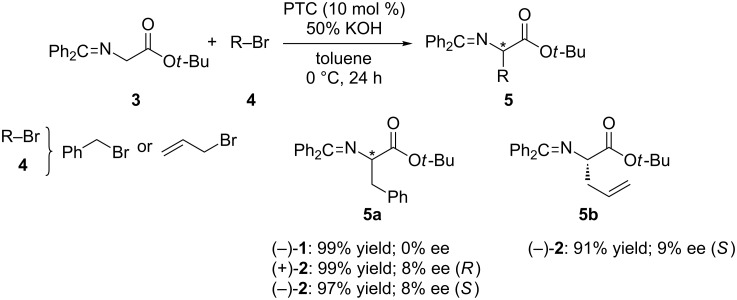
Asymmetric alkylations of **3** catalyzed by (±)-**1** and (±)-**2** under phase-transfer conditions.

In order to see the beneficial effect of the diarylmethanol structure in this catalysts, they were applied to the asymmetric alkylation of *tert*-butyl glycinate benzophenone Schiff base **3** with alkyl halides **4** in a toluene–50% KOH biphasic system (Scheme 1). The corresponding α-alkyl-α-amino acid derivatives **5** were obtained in excellent yields with very low enantioselectivities (up to 9%). This is the first example of asymmetric phase-transfer catalysis based on inherently chiral calix[4]arenes, although the asymmetric induction observed remained moderate.

**Scheme 2:**
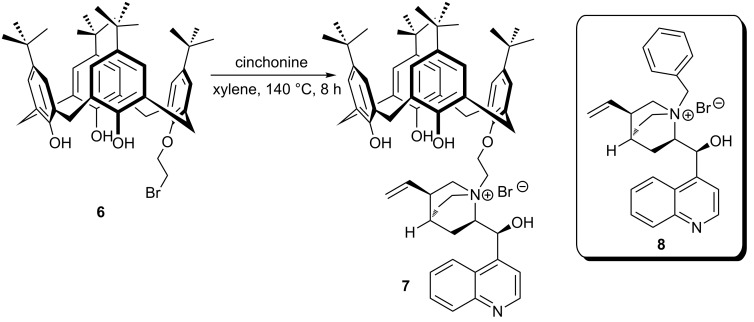
Synthesis of chiral calix[4]arene-based phase-transfer catalyst **7** and structure of O’Donnell’s *N*-benzylcinchonine **8**.

**Scheme 3:**
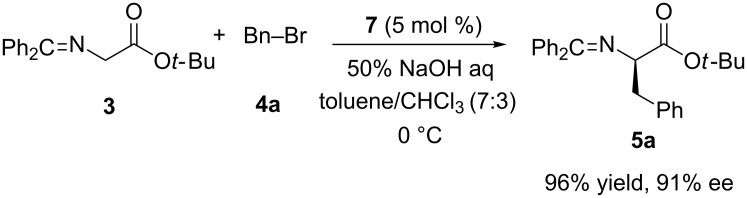
Asymmetric alkylation of glycine derivative **3** catalyzed by calixarene-based phase-transfer catalyst **7**.

Su et al. demonstrated a new approach for the design of a chiral binary integrative phase-transfer catalyst consisting of *p-tert*-butylcalix[4]arene and a cinchonine ammonium salt [38] (Scheme 2). Due to the failure of obtaining monobromo *p-tert*-butylcalix[4]arene derivative **6** directly from *p-tert*-butylcalix[4]arene using 1,2-dibromoethane in the presence of several bases, the synthetic route to calixarene-based chiral phase-transfer catalyst **7** comprises a four-step sequence including protection/deprotection steps for the benzyl groups. The catalytic efficiency of calix[4]arene-based phase-transfer catalyst **7** was evaluated in the benchmark reaction (Scheme 3) and compared with that of the chiral quaternary ammonium salt **8**. The results obtained when **7** used as asymmetric phase-transfer catalyst (APTC) in toluene/CHCl_3_–50% NaOH at 0 °C (96% yield, 91% ee) was better than both **8** or a mixture of calixarene and **8**. In the comparison experiments calix[4]arene and calix[6]arene were used as cocatalysts and it has been found that size of calixarene cavity played an important role in the catalytic activity and the selectivity. In addition, the catalytic performance of **7** was found sensitive to the cation of the base. Because, when aqueous NaOH was used instead of KOH, a higher yield and ee were obtained. It is proposed that a better chelating ability of **7** toward the sodium cation plays an important role in making the sodium enolate soluble in organic phase and this leads to enantiofacial differentiation in the transition state [39].

**Figure 2:**
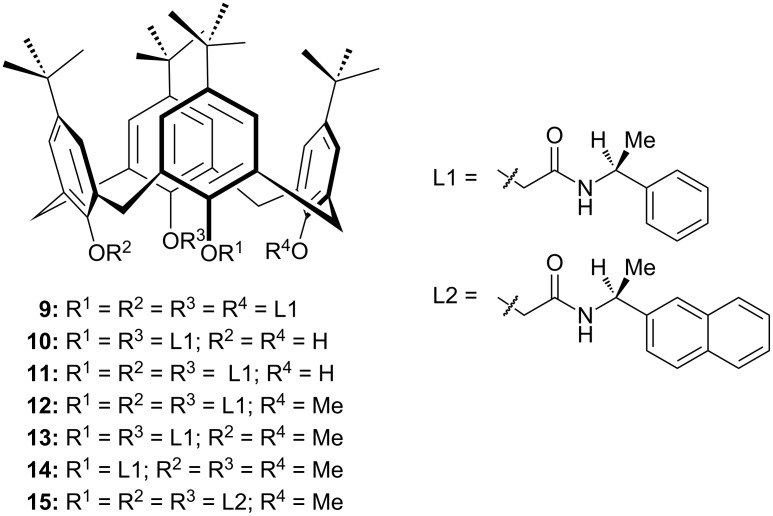
Calix[4]arene-amides used as phase-transfer catalysts.

**Scheme 4:**
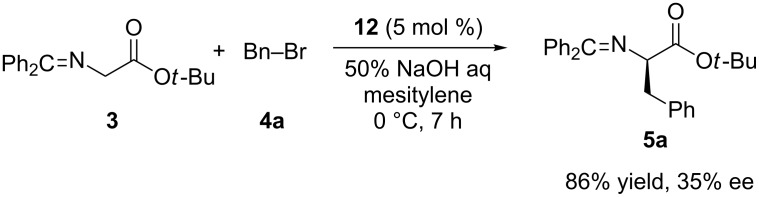
Phase-transfer alkylation of **3** catalyzed by calixarene-triamide **12**.

Just recently, Neri et al. utilized the cation recognition abilities of calixarene-amides in phase-transfer catalysis [40]. Seven chiral calix[4]arene derivatives **9–15** (Figure 2) bearing secondary amides at lower rim have been designed as catalysts and employed in the asymmetric alkylation of *N*-(diphenylmethylene)glycine esters. Among them, α-methylbenzylamine-derived calixarene-methoxy-triamide **12** afforded the (*R*)-benzylated product **5a** in 86% yield up to 35% ee (Scheme 4). In order to assess the effect of the calixarene backbone on the catalytic activity and enantioselectivity, they also performed the reaction in the presence of a chiral monoamide and an achiral calix[4]arene-tetramide. The results obtained in the presence of these catalysts confirmed the necessary role of the calixarene skeleton in phase-transfer catalysis by preorganizing and orienting the amide groups properly to favor the complexation of Na^+^ cations. The effect of the ester group in the substrate and other parameters on the reaction were also investigated. Under optimized conditions, up to 47% ee was obtained by alkylation of **3** with 4-methylbenzyl bromide in the presence of 5 mol % catalyst **12**.

### Henry reaction

**Scheme 5:**
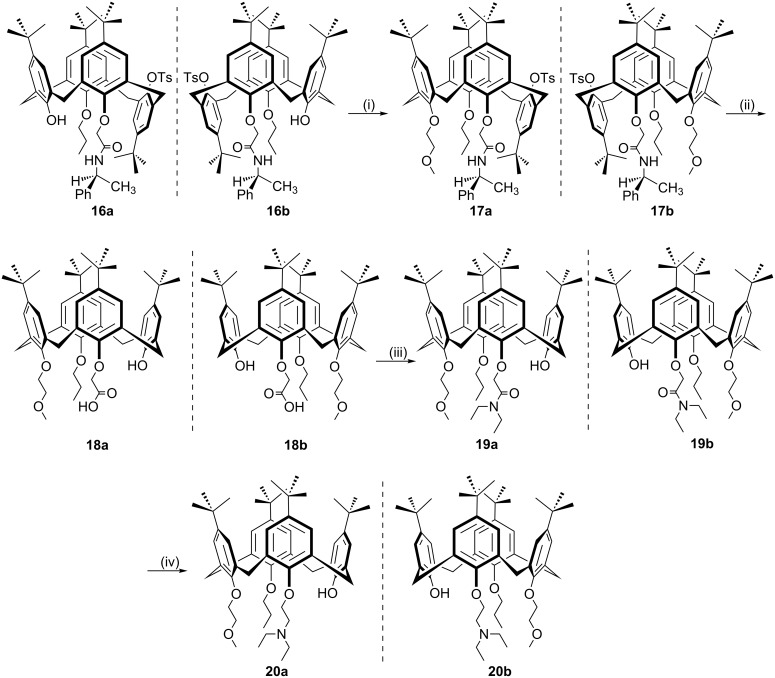
Synthesis of inherently chiral calix[4]arenes **20a/20b** substituted at the lower rim. Reaction conditions: (i) TsOCH_2_CH_2_OCH_3_/NaH, THF; (ii) *t*-BuOK, H_2_O/BuOH/DMSO; (iii) HNEt_2_, HBTU, CH_2_Cl_2_; (iv) LiAlH_4_/THF.

**Scheme 6:**
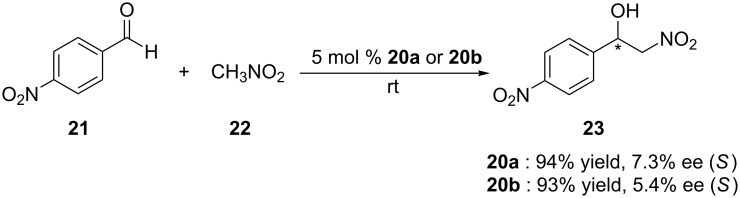
Asymmetric Henry reaction between **21** and **22** catalyzed by **20a/20b**.

**Figure 3:**
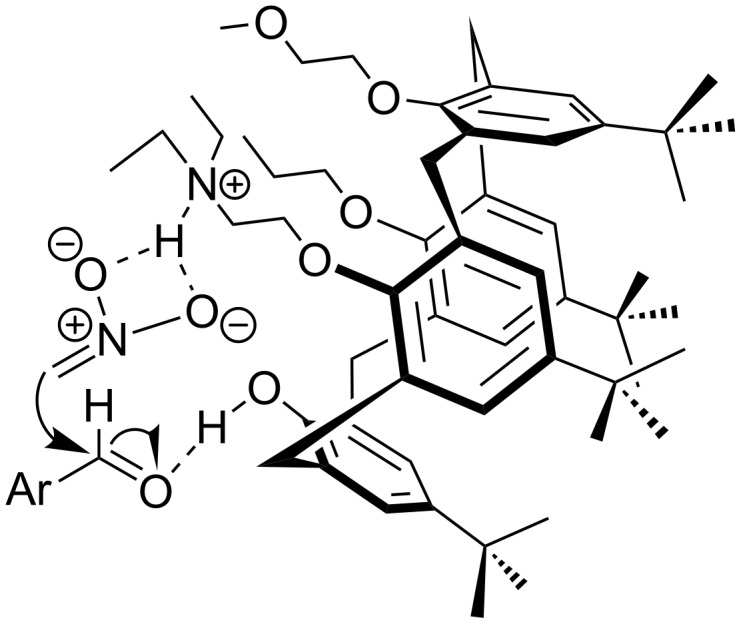
Proposed transition state model of asymmetric Henry reaction.

Since lower-rim functionalization of calix[4]arene is relatively easy, a variety of inherently chiral calix[4]arenes substituted at the lower rim have been successfully developed and used in chiral recognition. But their use in asymmetric organocatalysis hasn’t been reported. To explore the organocatalytic behaviors of inherently chiral calix[4]arenes modified at the lower rim, Li et al. reported the synthesis of *N,O*-type enantiomers based on inherently chiral calix[4]arene (Scheme 5) [41]. The synthetic sequence starts from a pair of optically pure inherently chiral calix[4]arene diastereomers **16a/16b** and contains four steps to lead **20a/20b** as a pair of enantiomers. The catalytic performance of these catalysts were then evaluated in the Henry (nitroaldol) reaction between 4-nitrobenzaldehyde (**21**) and nitromethane (**22**, Scheme 6). Excellent yields (up to 99%) were obtained in CH_3_CN and THF when H_2_O was added. The best enantioselectivity (7.5%) was achieved in a mixed protic solvent (EtOH/H_2_O, 3:1, v/v) with a yield of 54% when 5 mol % **20a** was used as catalyst. A dual activation model was proposed for the reaction (Figure 3). According to this mechanism, the aldehyde is activated by the phenolic hydroxy group of calix[4]arene through hydrogen bonding, while nitromethane is activated by tertiary amine group of the catalysts. The low enantioselectivities obtained were mainly attributed to the high flexibility of catalytic amino groups of *N,O*-type enantiomers.

### Suzuki–Miyaura cross–coupling and Tsuji–Trost allylic substitution reaction

**Scheme 7:**
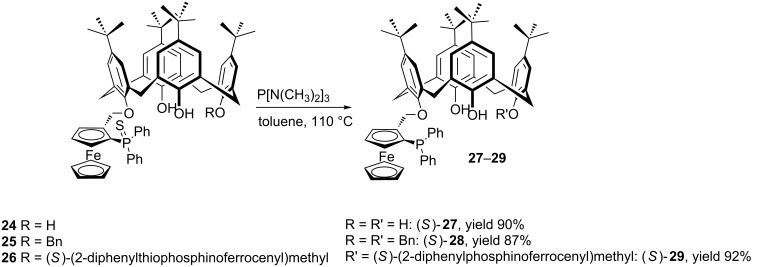
Synthesis of enantiomerically pure phosphinoferrocenyl-substituted calixarene ligands **27–29**.

Manoury et al. described the synthesis of ferrocene-bearing enantiomerically pure calixarenes and their catalytic performances in the asymmetric Suzuki–Miyaura coupling and Tsuji–Trost allylic alkylation reactions (Scheme 7) [42]. Calix[4]arene mono and dithiophosphines **24**–**26** were efficiently synthesized from *p-tert*-butylcalix[4]arene by a one pot Mitsunobu alkylation using enantiomerically pure (*S*)-(2-diphenylthiophosphinoferrocenyl)methanol. Deprotection of the thiophosphine unit(s) by tris(dimethylamino)phosphine gave chiral calixarene phosphines **27**–**29** bearing ferrocenyl substituents on the lower rim in high yields.

**Scheme 8:**
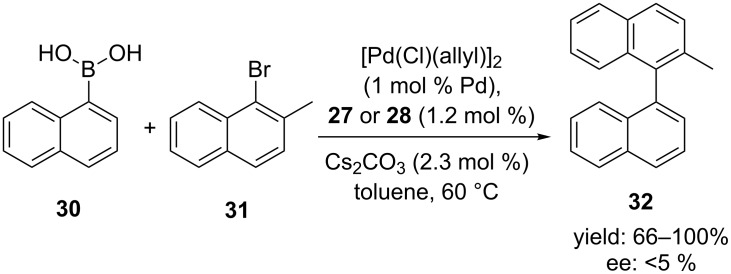
Asymmetric coupling reaction of aryl boronates and aryl halides in the presence of calixarene mono and di(ferrocenylphosphine) ligands **27** and **28**.

Calixarene-derived mono(ferrocenylphosphine) ligands **27** and **28** were tested in the palladium-catalyzed asymmetric Suzuki–Miyaura cross-coupling reaction for the first time in this study (Scheme 8). In order to see whether the calixarene backbone can effect the the coupling reaction between 1-naphthaleneboronic acid (**30**) and 1-bromo-2-methylnaphthalene (**31**), their reactions were compared to that of the model diphenylphosphino ferrocenes. When the allylpalladium chloride dimer was used as palladium precursor, good yields were obtained after 24 h. However, enantiomeric excesses for (*S*)-**27** and (*S*)-**28** (<5%) were lower than those obtained by using other phosphine-ether ligands based on the same chiral scaffold. It was concluded that free hydroxy groups on the lower rim of the calixarene ligands do not have a beneficial catalytic effect due to the formation of hydrogen bonds with the incoming boronic acids.

**Scheme 9:**
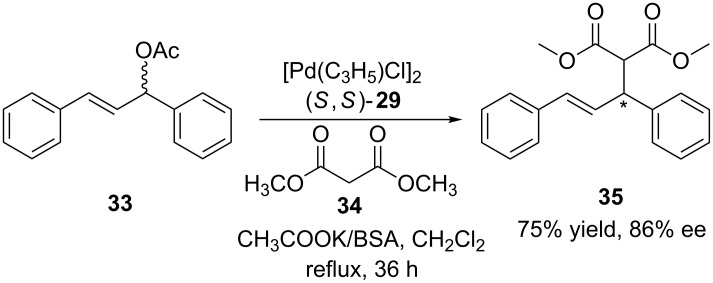
Asymmetric allylic alkylation in the presence of calix[4]arene ligand (*S*,*S*)-**29**.

In the same study, diphosphine ligand (*S,S*)-**29** was used in the asymmetric Tsuji–Trost allylic alkylation of 1,3-diphenylprop-2-enyl acetate (**33**) with dimethyl malonate (**34**, Scheme 9). Both, good catalytic activity and enantioselectivity (ee values up to 86%) were obtained with the potassium cation. This was due to the more strongly interaction of the dimethyl malonate anion with the two hydroxy groups of the calixarene ligand to selectively direct the nucleophile towards one carbon atom of the π-allyl intermediate in the presence of potassium cations.

**Figure 4:**
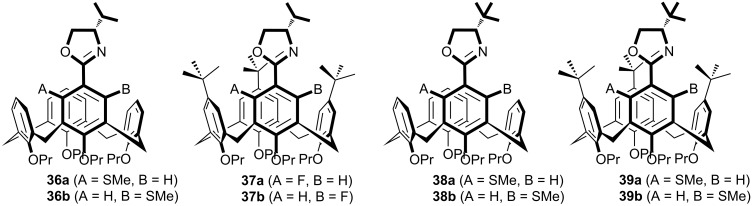
Structure of inherently chiral oxazoline calix[4]arenes applied in the palladium-catalyzed Tsuji–Trost allylation reaction.

**Scheme 10:**
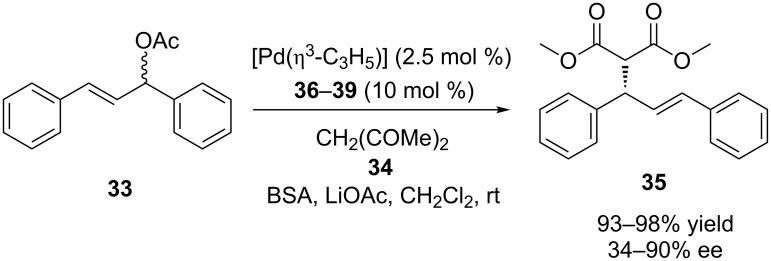
Asymmetric Tsuji–Trost reaction in the presence of calix[4]arene ligands **36–39**.

In 2009 and 2010, Arnott et al. utilized a chiral isopropyloxazoline as an *ortho*-lithiation directing group in order to synthesize inherently chiral calixarenes **36** and **37** [43–44]. In a continuation of their previous studies, they have reported diastereoselective synthesis of inherently chiral calixarene derivatives **38** and **39** via chiral *tert*-butyloxazoline-directed lithiation (Figure 4) [45]. It has been shown that selectivity of *ortho*-lithiation could be tuned by changing the alkyllithium reagent employed. The performance of four inherently chiral bidentate calix[4]arene ligands in the asymmetric Tsuji–Trost allylation reaction (Scheme 10) has been evaluated and compared to that of the planar model ligands. Inherently chiral calixarene ligands **36a/36b**–**38a/38b** were identified as being effective ligands not only for creation of adducts with excellent yields but also in reducing the reaction time compared to that of the ‘flat’ model ligands. From the results, it has been concluded that the source of the observed selectivity was the chiral oxazoline unit and not the inherent chirality of calixarene skeleton.

### Asymmetric hydrogenation

**Figure 5:**
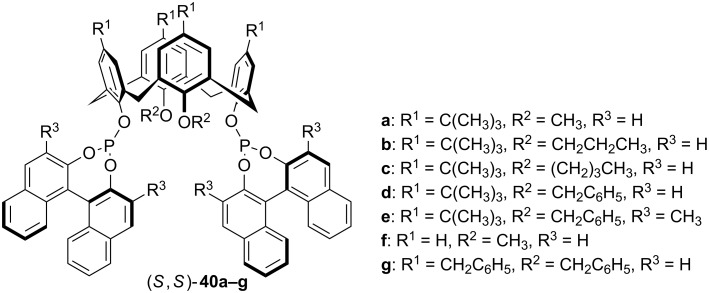
BINOL-derived calix[4]arene-diphosphite ligands.

Starting with distally *O*-dialkylated calixarene precursors, a series of BINOL-derived calix[4]arene-diphosphite ligands **40a–g** were synthesized by Liu and Sandoval through phosphorylation in the presence of NaH or *n*-BuLi (Figure 5) [46]. Conformational analysis of calixarene-diphosphite ligands revealed that presence of sterically hindered groups led to the formation of the predominantly cone-conformer.

**Scheme 11:**
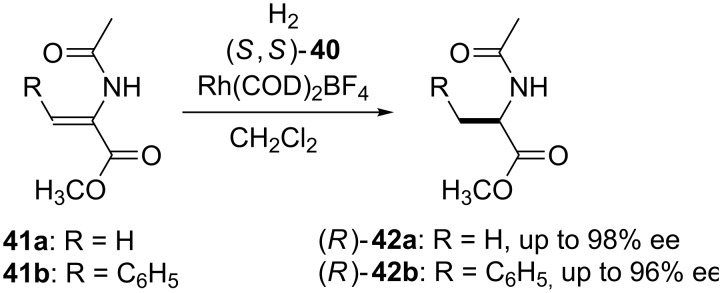
Asymmetric hydrogenation of **41a** and **41b** catalyzed by in situ-generated catalysts comprised of [Rh(COD)_2_BF_4_] and calix[4]arene-based chiral diphosphite ligands [(*S*,*S*)-**40**].

In order to check whether calix[4]arene-diphosphite ligands may also serve as efficient ligands in asymmetric catalytic reactions, the Rh-catalyzed asymmetric hydrogenation of methyl acetamidoacrylate (**41a**) and the corresponding cinnamate (**41b**) was evaluated in the presence of ligands **40a–g** (Scheme 11). The active catalyst was readily prepared in situ by mixing Rh(COD)_2_BF_4_ and corresponding (*S*,*S*)-**40** as obtained without further separation of conformational isomers. Both the catalytic activity and the stereoselectivity were changed to different extents depending to the substituents on the calixarene skeleton. Best efficiencies and enantioselectivities were obtained when conformationally rigid calixarenes were used. Hydrogenation of **41a** catalyzed by Rh/**40b** yielded (*R*)-**42a** quantitatively with 98% ee. The high catalytic activity of ligands **40** could be ascribed to combining of an effective bite-angle control with that of circular rigid platform.

### Michael addition

**Figure 6:**
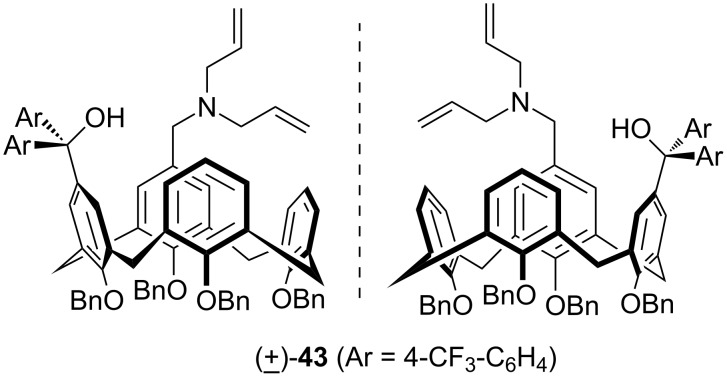
Inherently chiral calix[4]arene **43** containing a diarylmethanol structure.

**Scheme 12:**
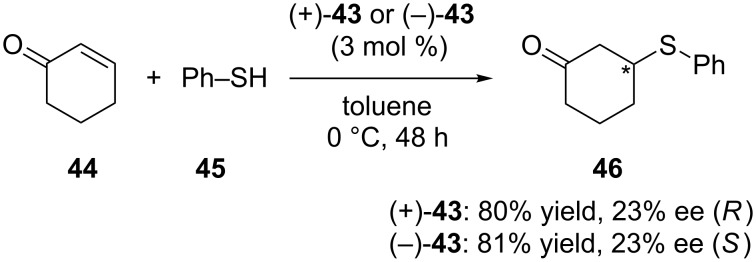
Asymmetric Michael addition reaction of **44** with **45** catalyzed by **43**.

It is known that the asymmetric Michael addition reaction of thiophenol could be catalyzed by inherently chiral calixarenes bearing amino alcohol/phenol structure [47–48]. In order to see the effect of the diarylmethanol moiety, Shirakawa and Shimizu used **43** (Figure 6) as organocatalyst in the Michael addition reaction between 2-cyclohexen-1-one (**44**) and thiophenol (**45**, Scheme 12) [37]. Compared to the inherently chiral calix[4]arene bearing an amino alcohol structure, a beneficial effect of the additional diaryl group was confirmed and the product was obtained in 23% ee with 81% yield. Substituted thiophenols, 2-cyclohepten-1-one and chalcone were also tested as substrates for the Michael addition reaction and the corresponding adducts were obtained in good yield with moderate enantioselectivity (13–23% ee).

**Figure 7:**
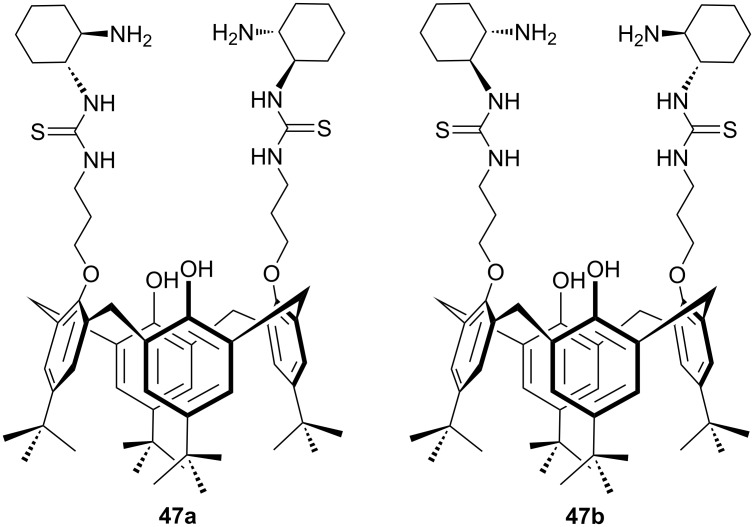
Calix[4]arene-based chiral primary amine–thiourea catalysts.

Calixarene-derived thiourea seemed to be an interesting backbone for design of new organocatalysts for asymmetric synthesis. In 2013, we described a novel class of calix[4]arene-based chiral primary amine–thiourea catalysts **47a** and **47b** derived from *p-tert*-butylcalix[4]arene (Figure 7) [49].

**Scheme 13:**
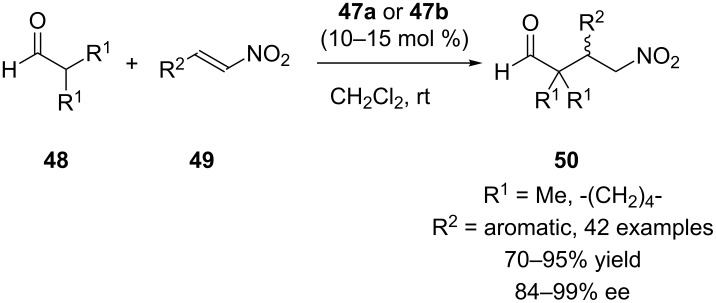
Asymmetric Michael addition of **48** with **49** catalyzed by **47a** and **47b**.

To evaluate the catalytic efficiency, bifunctional catalysts were applied to promote the Michael addition of aldehydes **48** with nitroalkenes **49** (Scheme 13). The asymmetric Michael addition in the presence of 10–15 mol % of the macrocyclic catalysts **47a/47b** afforded both enantiomers of the products **50** in high yields (up to 95%) and in high to excellent enantioselectivities (up to 99% ee). In order to confirm the role of the achiral calixarene platform, the monomeric analogue carrying both thiourea and primary amine subunits was also prepared and used in the control experiments. Under optimal reaction conditions, the noncyclic analogue afforded the product in lower yields and enantioselectivities (52% yield, 89% ee), which displayed the importance of the calix[4]arene skeleton.

**Scheme 14:**
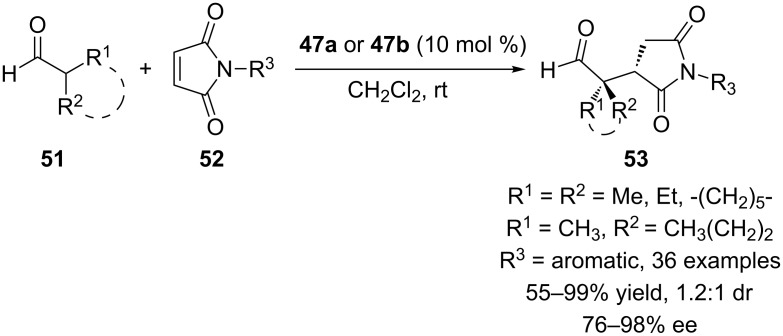
Enantioselective Michael addition of **51** to **52** catalyzed by calix[4]arene thioureas.

We also employed calixarene-based chiral primary amine thioureas **47a** and **47b** in the enantioselective Michael addition of α,α-disubstituted aldehydes **51** to maleimides **52** (Scheme 14) [50]. The reactions proceeded under mild conditions to give high yields (up to 99%) and ees (up to 98%) with broad substrate scope. The results indicated that the stereochemical outcome of the reaction was mainly governed by the 1,2-diaminocyclohexane moiety of thiourea and both enantiomers of the product could be obtained by altering the 1,2-diaminocyclohexane moiety from (1*R*,2*R*) to (1*S*,2*S*) without loss of activity or selectivity.

**Scheme 15:**
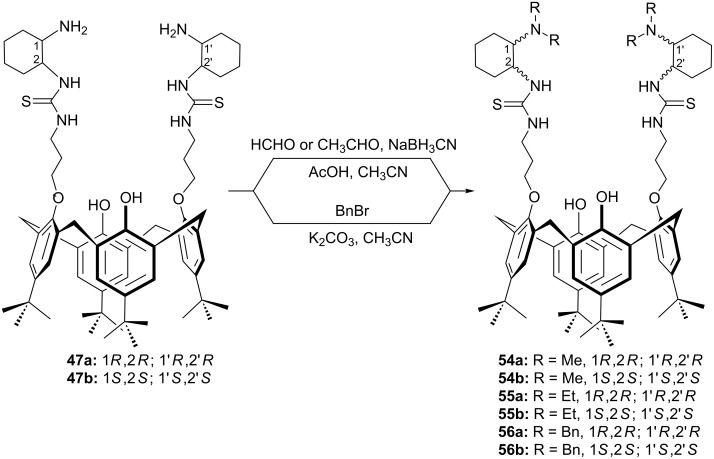
Synthesis of calix[4]arene-based tertiary amine–thioureas **54–56**.

Bifunctional organic molecules combining the thiourea moiety with a tertiary amine functionality are remarkably useful catalysts capable of simultaneous activation of both electrophiles and nucleophiles [51]. Therefore, a series of calixarene-based chiral bifunctional tertiary amine–thiourea organocatalysts **54–56** have been synthesized by us and Genc et al. (Scheme 15) [52–53].

**Scheme 16:**
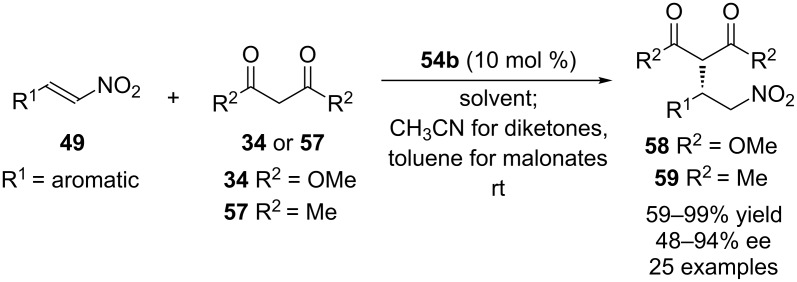
Asymmetric Michael addition of **34** and **57** to nitroalkenes **49** catalyzed by **54b**.

Among them, **54a**,**b** were applied to the asymmetric Michael addition of 1,3-dicarbonyl compounds **34** and **57** to a variety of nitroolefins. Although both catalysts gave the Michael adduct in excellent yields, high ees were obtained only when **54b** was used as organocatalyst (up to 94% ee, Scheme 16).

**Scheme 17:**
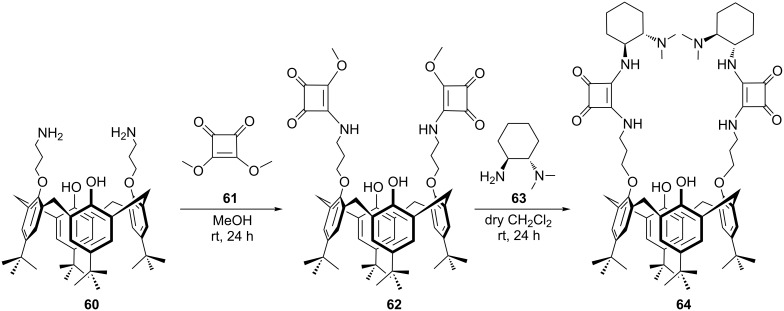
Synthesis of *p-tert*-butylcalix[4]arene bis-squaramide derivative **64**.

During the last decade, squaramide catalysts have become a powerful alternative to the urea/thiourea and guanidine catalysts as multiple hydrogen bond donors in order to design novel bifunctional catalytic scaffolds [54–55]. Hybrid calixarene hosts bearing bis-squaramide moieties at the *endo* (or lower) and *exo* (or upper) rims and their recognition properties toward anionic guests have been already reported [56–57]. Therefore, it would be interesting to investigate the application of chiral calixarene-based squaramides in asymmetric catalysis. Recently, a novel chiral organocatalyst based on the calix[4]arene scaffold carrying squaramide and tertiary amine as catalytic functionalities has been readily developed in two steps from *p-tert-*butylcalix[4]arene diamine **60** in our lab [58]. Accordingly, dimethyl squarate **61** was stirred with *p-tert*-butylcalix[4]arene diamine **60** in CH_2_Cl_2_ to give squarate **62**. Subsequently, **62** was treated with (1*S*,2*S*)-*N*,*N*-dimethyl-1,2-diaminocyclohexane (**63**) under mild conditions, furnishing the final catalyst **64** (Scheme 17).

**Scheme 18:**
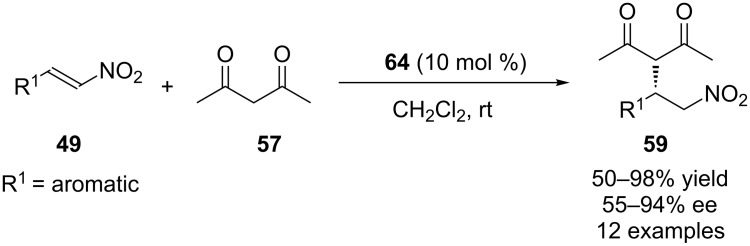
Asymmetric Michael addition catalyzed by **64**.

Asymmetric Michael addition of **57** to **49** catalyzed by **64** at room temperature afforded the Michael adducts in high yields and moderate to excellent enantioselectivities (Scheme 18).

**Scheme 19:**
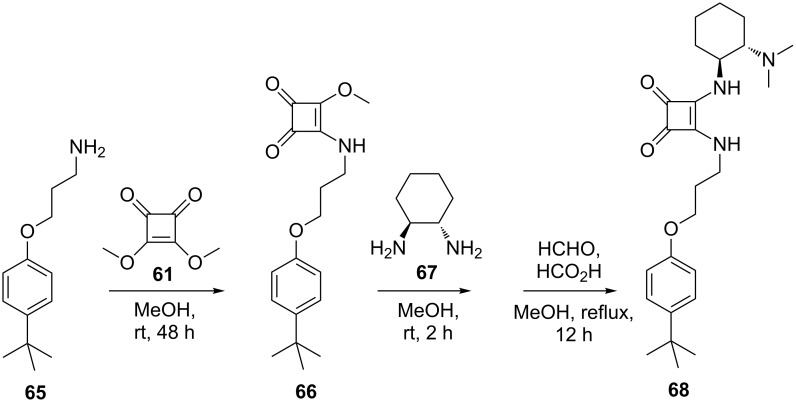
Synthesis of chiral *p-tert*-butylphenol analogue **68**.

To establish the role of the calixarene backbone on the enantioselective Michael addition, chiral monomeric analogue **68** bearing the same bifunctional pattern was also prepared from *p-tert*-butylphenol and used for comparison (Scheme 19). When monomeric analog **68** was used as a catalyst alone or in the presence of *p-tert*-butylphenol/phenol as acidic additives, lower yield and enantioselectivity were observed. These results clearly confirmed the cooperative effect and special role of the calixarene backbone.

### Aldol reaction

**Figure 8:**
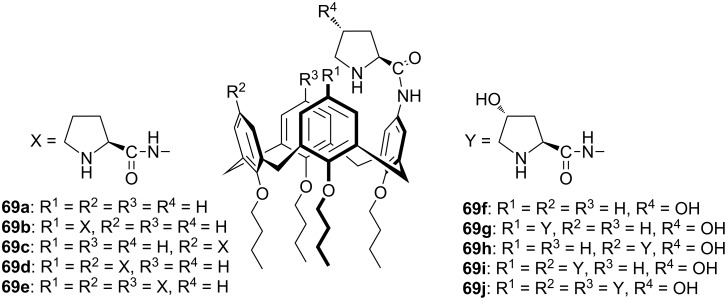
Novel prolinamide organocatalysts based on the calix[4]arene scaffold.

Since chiral substituents and binding sites attached to the upper rim of the calixarene backbone can offer the full advantage of the unique inclusion properties of hydrophobic cavity, Wang et al. reported the synthesis of a series of prolinamide and hydroxyprolinamide organocatalysts based on the calix[4]arene scaffold (Figure 8) [59]. Treatment of Boc-protected-L-proline or hydroxyproline with various aminocalix[4]arenes under one of the appropriate coupling conditions and subsequent deprotection of Boc group gave the target compounds **69**.

**Scheme 20:**
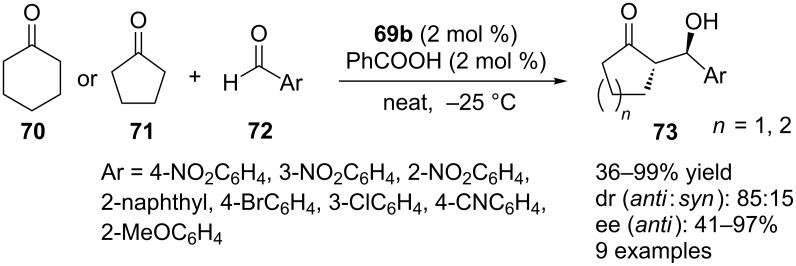
Asymmetric aldol reactions of **72** with **70** and **71** catalyzed by **69b**.

The catalytic activities of these prolinamide organocatalysts based on the calix[4]arene scaffold were evaluated for the model reaction between **21** and **70**. The results showed that under solvent-free conditions at −25 °C, 2 mol % catalyst **69b** with two prolinamide units located appropriate array on calixarene in the presence of 2 mol % of PhCOOH catalyzed the aldol reaction in excellent yield and high enantioselectivity. Under the optimized reaction conditions, the aldol reaction of aromatic aldehydes **72** with **70** and **71** afforded the adducts in moderate to good yields (up to >99%) with up to 97% ee and up to 85:15 dr (Scheme 20).

**Scheme 21:**
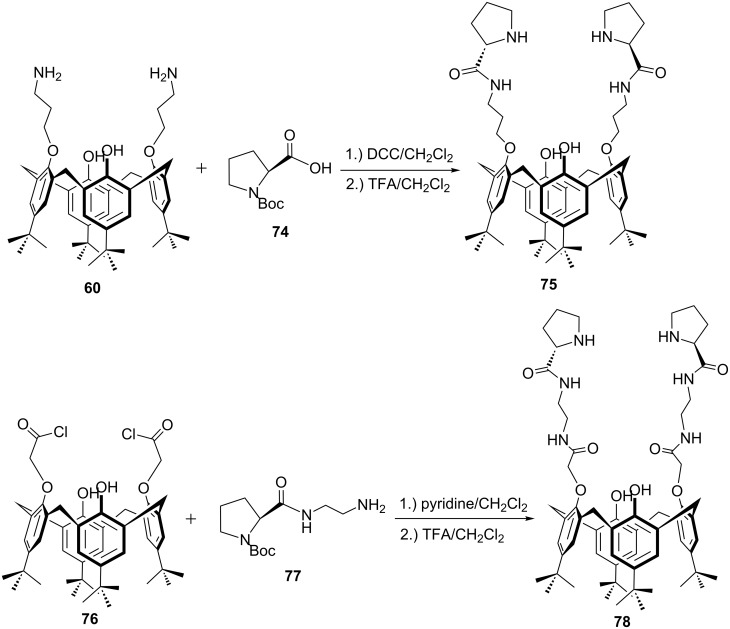
Synthesis of *p-tert*-butylcalix[4]arene-based chiral organocatalysts **75** and **78** derived from L-proline.

In order to exploit the hydrophobic cavity of the calixarene platform in aldol reactions, two novel *p-tert*-butylcalix[4]arene-based chiral organocatalysts bearing L-proline units on the lower rim have been reported by Yilmaz et al. [60]. Coupling of calixarene diamine **60** with Boc-L-proline **74** or calixarene diacid chloride **76** with *N*-(2-aminoethyl)-*N'*-(*tert*-butoxycarbonyl)-L-prolinamide **77** and subsequent deprotection afforded catalysts **75** and **78** in good yields (Scheme 21).

**Scheme 22:**
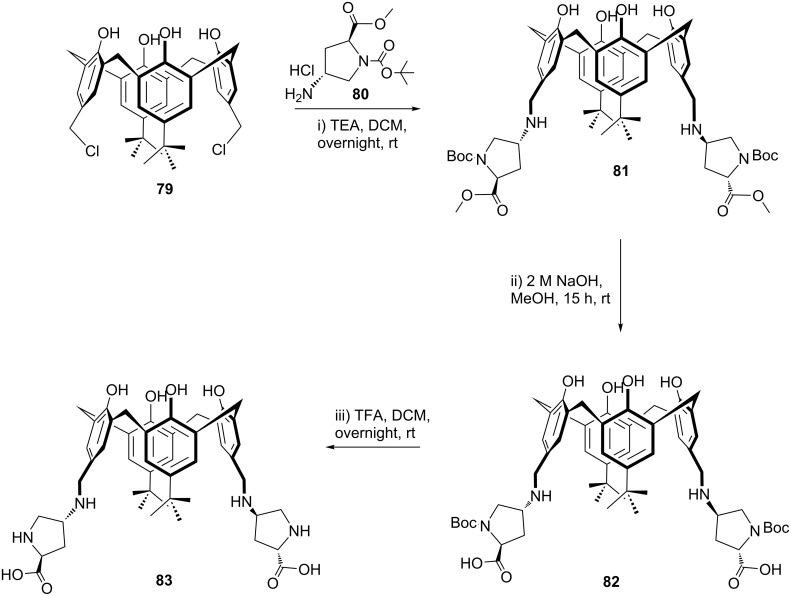
Synthesis of upper rim-functionalized calix[4]arene-based L-proline derivative **83**.

In 2014, an upper rim functionalized calix[4]arene-based L-proline derivative has been reported [61]. As shown in Scheme 22, firstly chloromethylated compound **79** was reacted with *N*-Boc-*trans*-4-amino-*L*-proline methyl ester hydrochloride **80** in the presence of triethylamine to give the corresponding Boc-protected proline methyl ester of **81**, which was then converted to the the carboxylic acid analogue **82** by treatment with sodium hydroxide in MeOH at ambient temperature. Finally, deprotection of Boc groups under standard conditions afforded the targeted organocatalyst **83**.

**Scheme 23:**
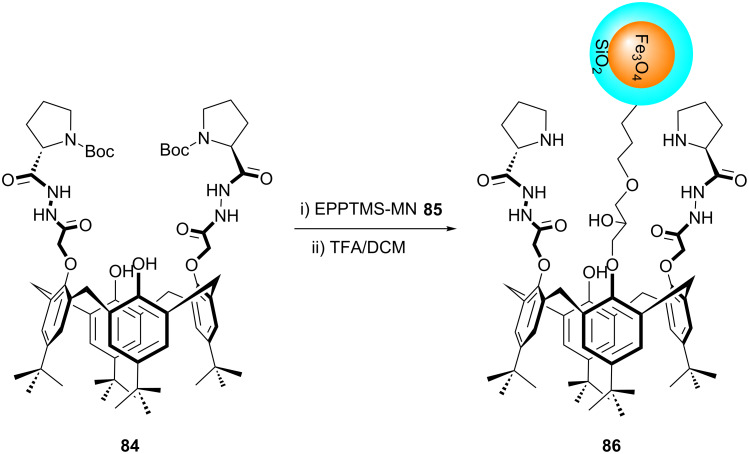
Synthesis and proposed structure of Calix-Pro-MN (**86**).

Magnetic nanoparticles (MNPs) due to their high surface area have been widely used as catalytic supports. Surface modification of MNPs with chiral organocatalysts for asymmetric catalysis provides sustainable materials which could perform chiral transformations robustly and readily [62–63]. In this context, the use of chiral calixarenes is very limited. For the first time, a chiral calixarene derivative functionalized with two proline units on the lower rim was supported onto well defined (15 ± 3 nm) magnetic Fe_3_O_4_ nanoparticles by Yilmaz et al. [64]. Reaction of compound **84** with [3-(2,3-epoxypropoxy)propyl]trimethoxysilane-modified Fe_3_O_4_ nanoparticles (EPPTMS-MN) **85** in the presence of sodium hydride in tetrahydrofuran and subsequent deprotection of Boc groups afforded L-proline immobilized calix[4]arene magnetic nanoparticles (Calix-Pro-MN) **86** (Scheme 23).

**Figure 9:**
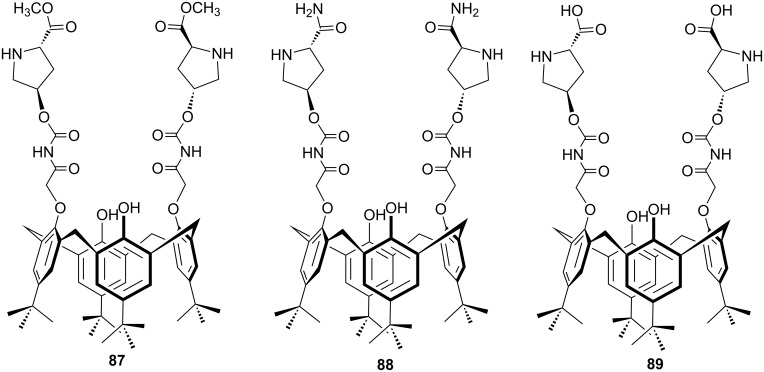
Calix[4]arene-based L-proline catalysts containing ester, amide and acid units.

In 2016, three new lower rim functionalized calix[4]arene-based L-proline catalysts **87–89** containing ester, amide and acid units respectively have been designed by Yilmaz et al. as shown in Figure 9 [65].

**Scheme 24:**
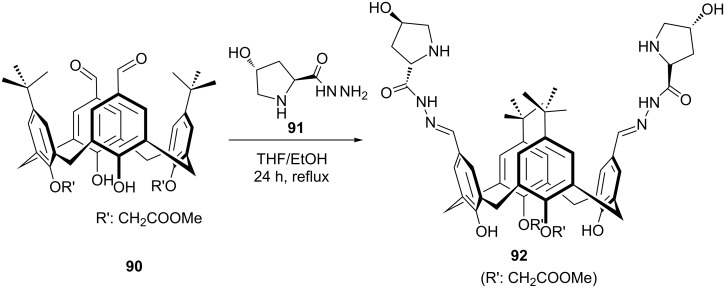
Synthesis of calix[4]arene-based prolinamide **92**.

A new prolinamide derivative **92** with increased NH acidity has been synthesized from diformylcalixarene derivative **90** as shown in Scheme 24 [66].

**Scheme 25:**
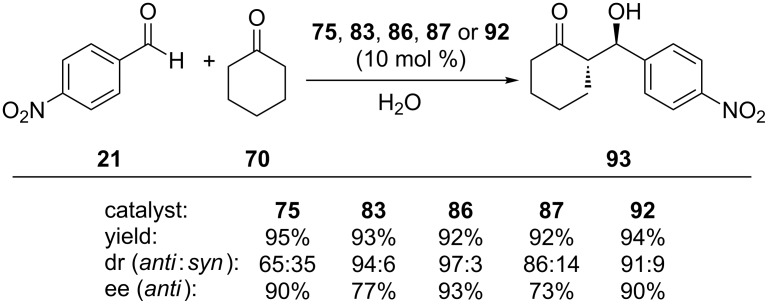
Calixarene-based catalysts for the aldol reaction of **21** with **70**.

**Scheme 26:**
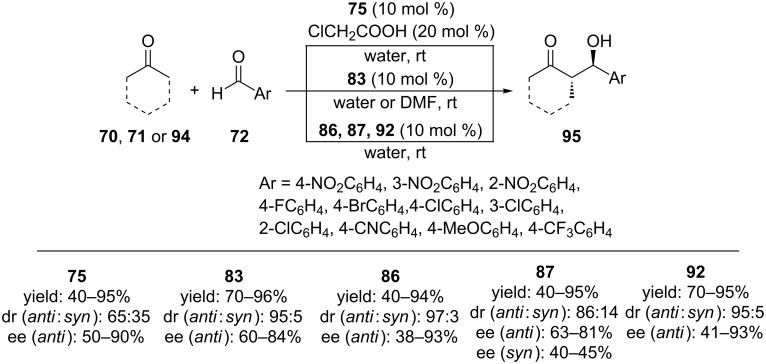
Asymmetric aldol reactions of **72** with cyclic ketones catalyzed by calix[4]arene-based chiral organocatalysts derived from L-proline.

Aldol reaction between **21** and **70** in water showed that catalyst **75** provided faster reaction times and higher isolated yields compared to those of catalyst **78**. When 10 mol % of **75** in the presence of 20 mol % ClCH_2_COOH in water was employed for the model reaction, 95% yield, 90% ee and 65:35 dr were obtained (Scheme 25). Moreover, a monomeric analogue without calixarene skeleton was also employed and found to be completely ineffective (<10% conversion, 56:42 dr), indicating that the hydrophobic calixarene platform was crucial. Aromatic aldehydes with different substitution patterns on the benzene ring were also tested as aldol acceptors and high yields (up to 95%), enantioselectivities (up to 90%) but moderate diastereoselectivities (up to 65:35) were obtained (Scheme 26). A general organocatalyzed aldol reaction mechanism in water was suggested by the authors. Accordingly, the observed catalytic activity and stereoselectivity was explained by the formation of a hydrophobic and hydrophilic region via hydrogen bonds between **75** and interfacial water molecules.

The catalytic activity of compound **83** as an organocatalyst was evaluated in the enantioselective aldol reaction of **21** and **70** in different amounts of water. In the presence of 10 mol % catalyst, the aldol adduct was obtained in 99% conversion, 94:6 dr and 77% ee in 0.25 mL water. Under the optimized reaction conditions, the substrate scope for **83**-catalyzed aldol reactions were also probed in DMF. Various aromatic aldehydes reacted well with **70** to give the aldol products in excellent yields with good ees. Particularly, nitro-substituted benzaldehydes gave the best enantioselectivity, and the desired aldol products were obtained with *anti* diastereoselectivity (Scheme 26). In addition, when DMF was used as solvent, a positive effect on the enantioselectivity but negative effect on the diastereoselectivity were observed.

The catalytic activity of Calix-Pro-MN **86** was also tested on the asymmetric aldol reactions in water (without organic solvents) and almost full conversion of substrates into products with high enantioselectivity and diastreoselectivity were achieved. In the substrate scope studies, aromatic aldehydes bearing electron-withdrawing groups at the 4-position of the benzene ring reacted smoothly to give the corresponding products in high yields with high enantioselectivities (up to 93% ee) and diastereoselectivities (up to 97:3, *anti*/*syn*) (Scheme 26). The aldol reaction between **21** and **71** afforded mainly the *syn*-aldol product in 90% yield with 70% ee. The catalyst could readily be isolated by applying an external magnetic field and recycled five times without significant loss of activity.

Among structurally similar calixarene-based L-proline catalysts **87**–**89**, compound **87** was found to be effective in the model aldol reaction between **21** and **70** and offered superior diastereo- and enantioselectivities in the presence of chloroacetic acid (96% conversion, 86:14 *anti* diastereoselectivity and 73% ee) (Scheme 25). Under the optimum reaction media, a series of diverse benzaldehydes bearing electron withdrawing groups were reacted with **70**. Among them, the reactions of **70** with *o*-nitrobenzaldehyde and *o*-chlorobenzaldehyde provided the best ees (up to 81% ee) and diastereoselectivities (up to 90:10 dr). Aldol reaction of **21** and **71** in the presence of catalyst **86** resulted in the formation of *syn*-aldol product as the major diastereomer with moderate yield but the selectivities were low. Acetone (**94**) was also used as substrate but only 40% ee was obtained although the yield was 80% (Scheme 26).

In order to create a more compact transition state and increase the selectivity by this way in direct stereoselective aldol reactions, optically active calix[4]arene-imine derivative **92** containing an L-prolinamide functionality was also tested as a catalyst for enantioselective aldol reactions between **70** and a variety of aromatic aldehydes **72**. The *anti*-aldol adducts were obtained in high yields and ees (Scheme 26).

**Figure 10:**
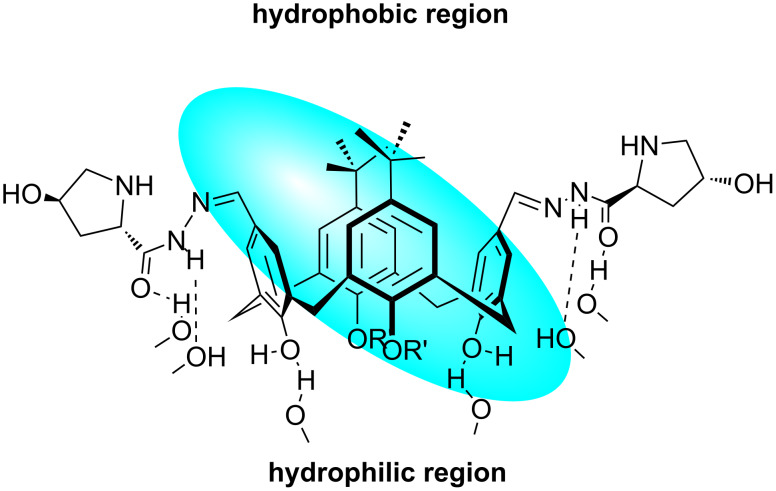
A proposed structure for catalyst **92** in H_2_O.

Based on the results, a plausible reaction mechanism similar to that previously suggested for the L-proline-catalyzed aldol reactions in water which proceeds via an enamine intermediate was proposed. As shown in Figure 10, it is proposed that hydrogen bonds between the OH and NH groups of the calix[4]arene catalyst and water molecules led to formation of hydrophobic and hydrophilic regions. This in situ-formed system enhanced the activity and selectivity of catalyst **92**. The Houk–List transition state model was suggested to account for the observed high selectivity.

**Scheme 27:**
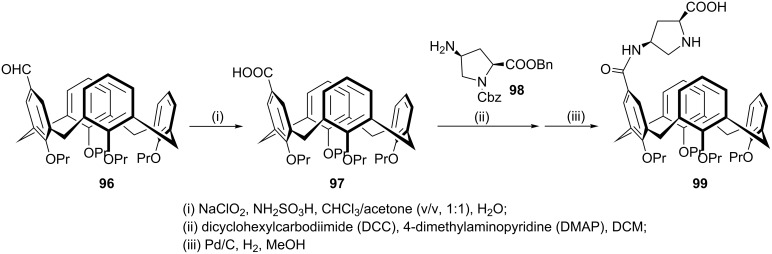
Synthetic route for organocatalyst **98**.

**Scheme 28:**
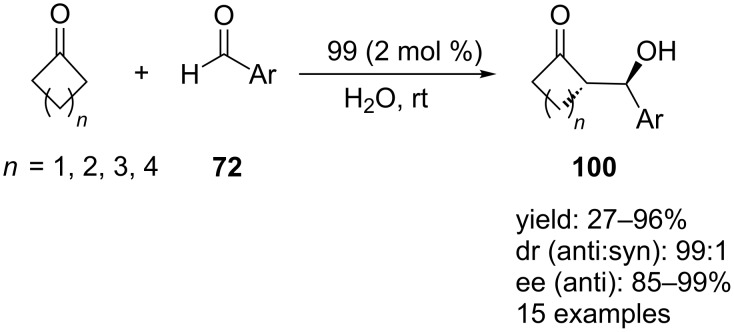
Asymmetric aldol reactions catalyzed by **99**.

Just recently, design and synthesis of a series of upper-rim-functionalized calix[4]arene-based L-proline derivatives have been reported by Sun et al. [67]. Among them, the chiral calixarene catalyst **99** which was prepared starting from 5-formyl-25,26,27,28-tetrapropoxycalix[4]arene **96** in three steps (oxidation to **97** then condensation with (2*S*,4*S*)-4-aminopyrrolidine-1,2-dicarboxylate **98** under standard coupling conditions and final deprotection, Scheme 27) showed excellent catalytic activity towards the model reaction between **21** and **70.** Under optimum conditions the adduct was formed in 96% yield, 97:3 dr as well as 99% ee in the presence of water. The high catalytic efficieny of this proline-functionalized calix[4]arene organocatalyst was attributed to the presence of the amide group and its synergistic effect through hydrogen-bonding interaction with the substrate. The scope and limitations of the asymmetric aldol reactions between a wide range of aromatic aldehydes and cyclic ketones with different sizes were examined (Scheme 28). Generally, the aldol adducts were provided in good to excellent yields, diastereoselectivities and enantioselectivities. The aromatic aldehydes bearing strong electron-withdrawing groups mainly gave *anti*-aldol products with high ee. In addition, the present method demonstrated selectivity towards cyclic ketones. According to the results observed, cyclic ketones of five- and six-membered sizes evolved into intermediates which show ideal affinity to the calixarene cavity.

**Figure 11:**
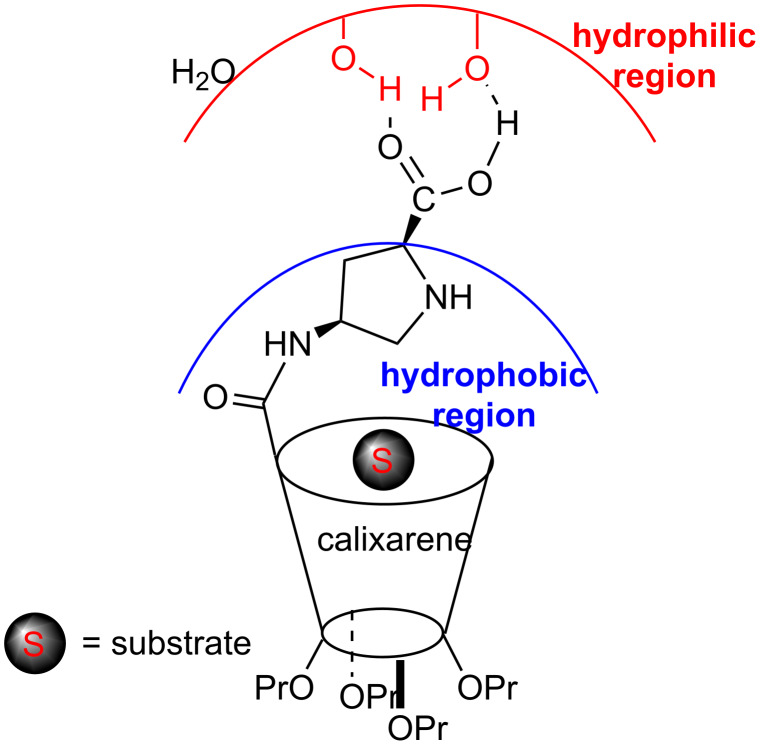
Proposed catalytic environment for catalyst **99** in the presence of water.

A mechanism based on general organo-mediated aldol reactions in H_2_O was proposed for the present methodology. Accordingly, a hydrophobic region and a hydrophilic region may be created through the hydrogen bonds between functionalized calix[4]arene and water molecules. This leads to in situ formation of a microreactor that improves the activity and stereoselectivity of catalyst **99**. As shown in Figure 11, this catalytic system renders to use the calixarene cavity and in consequence of this both the high diastereoselectivity and enantioselectivity can be explained. Furthermore, when **99** used in 2 mol % can be recycled easily and re-used six times with slightly decrease in its yields and diastereoselectivities.

**Scheme 29:**
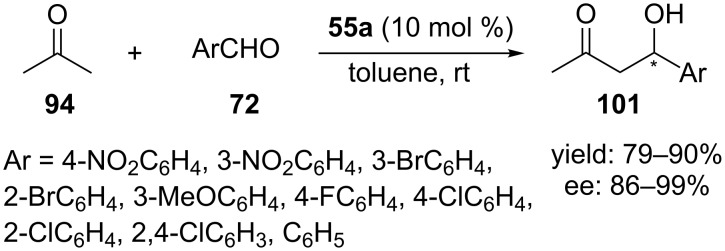
Asymmetric aldol reactions between **94** and **72** catalyzed by **55a**.

Genc et al. reported that the calixarene-based chiral tertiary amine–thioureas **55** and **56** (Scheme 15) could be used as catalyst in the asymmetric aldol reactions between **94** and **72** [53]. The aldol adducts were obtained in 79–90% yield with up to 99% ee using 10 mol % of catalyst **55a** in toluene after 3–5 days (Scheme 29). Although the authors did not propose a plausible mechanism, it was clear in this tertiary amine-thiourea-catalyzed aldol reactions formation of an enamine intermediate was not involved. Whether the reaction works through the enolate mechanism and complete noncovalent catalysis still needs further investigation.

### Biginelli reaction

**Scheme 30:**
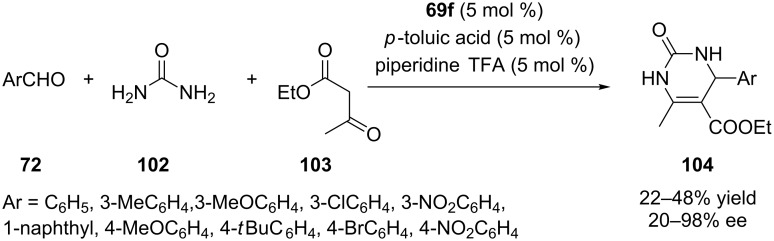
Enantioselective Biginelli reactions catalyzed by **69f**.

The catalysts **69f–j** (Figure 8) were successfully applied to the enantioselective multicomponent Biginelli reaction of benzaldehyde, ethyl acetoacetate and urea [68]. The results show that all five chiral calix[4]arene catalysts promote the formation of the dihydropyrimidine product with different extents in terms of chemical yield and enantioselectivity. For the model reaction the best enantioselectivity (54% ee) was obtained when mono-hydroxyprolinamide-based calix[4]arene **69f** was used as catalyst. This result could be ascribed to the effect of steric hindrance on the upper rim of calix[4]arene. So, the optimization and substrate scope studies were performed by using chiral catalyst **69f** and up to 98% ee was obtained (Scheme 30). In order to confirm the role of the calixarene skeleton, an L-prolinamide analogue was prepared as a model catalyst from *p*-butoxybenzenamine. Without calix[4]arene backbone, only 9% ee was obtained. It was also proposed that the observed enantioselectivity resulted from the approach of the enamine double bond to the *Re* face of the imine via a stable six-membered-ring transition state similar to that described earlier by Feng et al. [69].

### Epoxidation

**Scheme 31:**
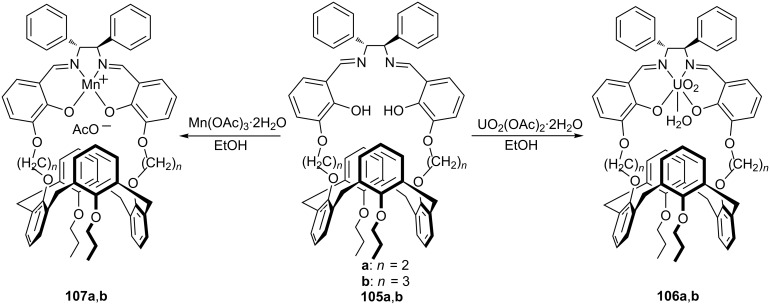
Synthesis of calix[4]arene–(salen) complexes.

**Scheme 32:**
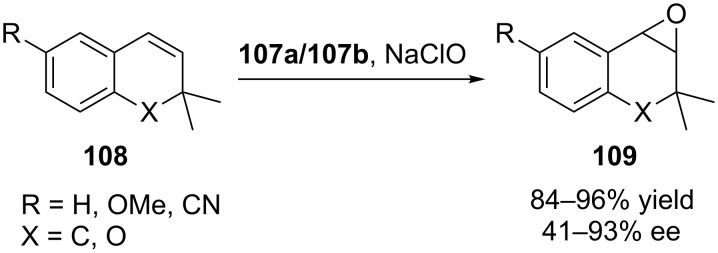
Enantioselective epoxidation of **108** catalyzed by **107a**/**107b**.

In 2014 Sciotto et al. reported the synthesis of two novel calix[4]arene–salen ligands **105a**,**b** in 1,3-alternate conformation. Reaction of the salen ligands with appropriate metal acetate salt according to the Scheme 31, led to the formation of uranyl and manganese complexes **106a**,**b–107a**,**b** [70]. While Mn(III) complexes **107a**,**b** have been used as catalysts for asymmetric epoxidation of styrene and substituted styrenes in the presence of NaClO as an oxygen donor and 4-phenylpyridine *N*-oxide (4-PPNO) as a coligand, their uranyl-(salen) derivatives **106a**,**b** have been employed as models of the oxo-Mn(V)–(salen) oxidant species. The reactions were highly efficient in terms of productivity (up to 96% yield) and enantioselectivity (up to 93% ee) when rigid bicyclic alkenes such as 1,2-dihydronaphthalene and substituted 2,2’-dimethylchromene were used as substrates (Scheme 32). It was worth to note that for all the studied alkenes, catalyst **107a** afforded higher ee values than catalyst **107b** and this was ascribed to the length of the methylene spacers and more rigid structure of **107a**.

### Meerwein–Ponndorf–Verley (MPV) reduction

**Scheme 33:**
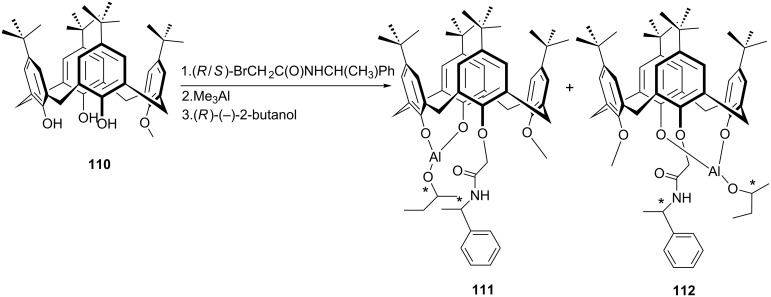
Synthesis of inherently chiral calix[4]arene catalysts **111** and **112**.

**Scheme 34:**
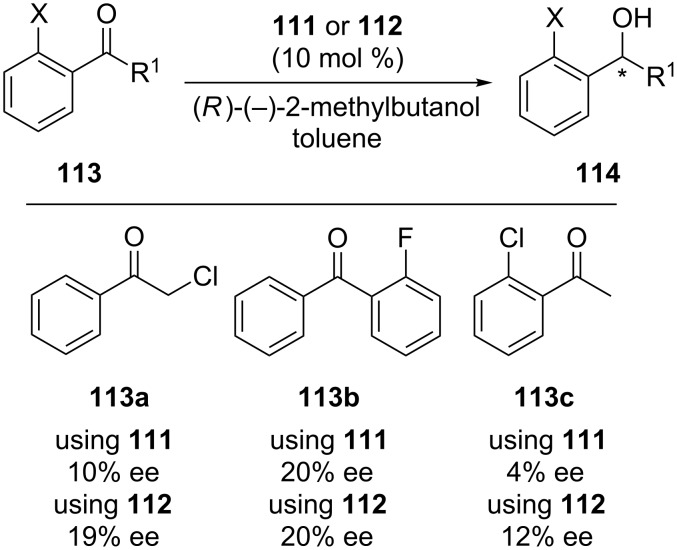
Enantioselective MPV reduction.

In 2011, Katz et al. developed two optically pure aluminum complexes of inherently chiral calix[4]arene **111** and **112** bearing also an asymmetric carbon center on the phenylethylamine substituent (Scheme 33) as catalysts for asymmetric Meerwein–Ponndorf–Verley (MPV) reduction of acetophenone derivatives using *R*-(−)-2-methylbutanol as hydride source [71]. As shown in Scheme 34, independent of which calix[4]arene diastereomer (**111** or **112**) was employed, when *ortho*-fluorobenzophenone was used as substrate, the enantioselectivity remained at 20%. But the MPV enantioselectivity was found sensitive to the inherent chirality of calix[4]arene for α-chloroacetophenone and *ortho*-chloroacetophenone. The results clearly showed the effect of both denticity of ketone reactant and cooperativity between normal and inherent chirality on the selectivity.

**Scheme 35:**
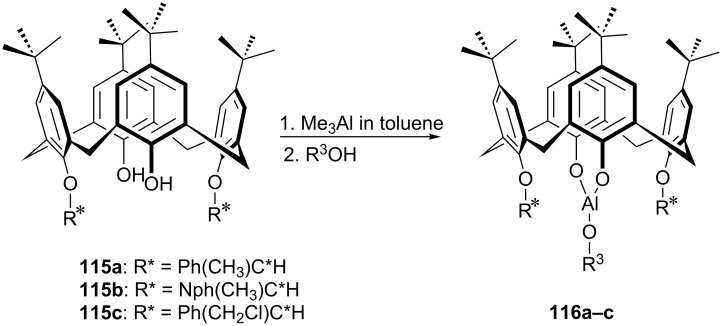
Synthesis of chiral calix[4]arene ligands **116a–c**.

**Scheme 36:**
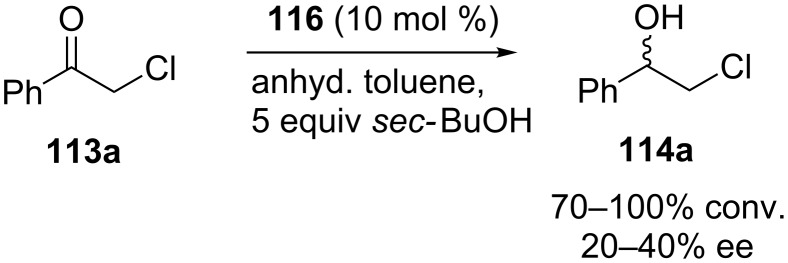
Asymmetric MPV reduction with chiral calix[4]arene ligands.

More recently, Al(III)–calix[4]arene complexes **116a–c** have been prepared from enantiopure chiral hemispherical calix[4]arene ligands **115a–c** in which the asymmetric carbon is directly attached to the calixarene lower rim as shown by Katz et al. (Scheme 35) for the asymmetric Meerwein−Ponndorf−Verley (MPV) reduction reaction [72]. Chiral *sec*-butylalcohol was used as a hydride donor. The data for MPV reduction of **113a** at room temperature showed that the enantioselectivity was highly dependent on the steric bulk of catalyst (Scheme 36). Enantioselectivity of the reduction increased up to 20% when lower-rim substituent changed from α-phenylmethyl in **115a** to α-naphthylmethyl in **115b**. To increase the enantioselectivity further, specific dative interactions between the Al(III) site and a Cl substituent on the lower rim of ligand **115c** were also exploited and 40% ee was obtained for the same model reaction.

**Scheme 37:**
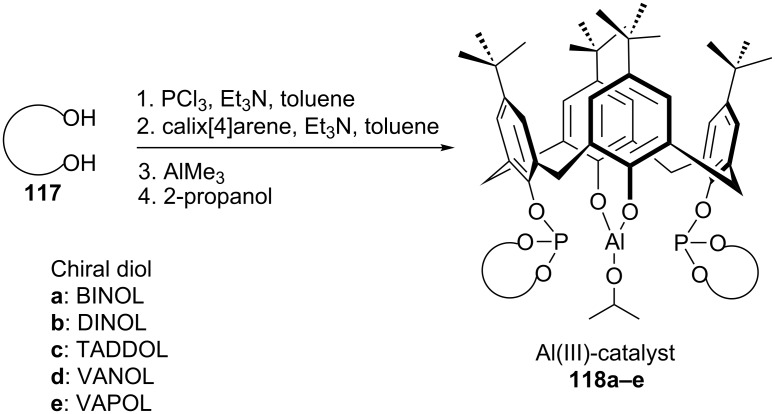
Chiral Al^III^–calixarene complexes bearing distally positioned chiral substituents.

**Scheme 38:**
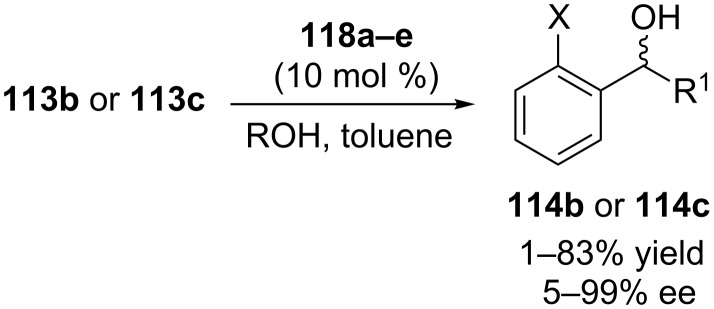
Asymmetric MPV reduction in the presence of chiral calix[4]arene diphosphites.

Inspired by this enantioselectivity increase due to the noncovalent interactions, aluminum complexes of calixarene diphosphites **118a–e** were synthesized via lower-rim functionalization of the *p-tert*-butylcalix[4]arene core with axially chiral diol ligands **117** (Scheme 37). The asymmetric MPV reduction of various substituted acetophenones were performed using **118a–e** as catalysts and isopropanol as a secondary alcohol hydride donor (Scheme 38). Although 99% ee was achieved with catalyst **118a** in initial conversion stage, from the perspective of yield and enantioselectivity VANOL-derived phosphite catalyst **118d** which has an extended π-delocalization exhibited the best result.

### Aza-Diels–Alder and epoxide ring-opening reaction

**Scheme 39:**
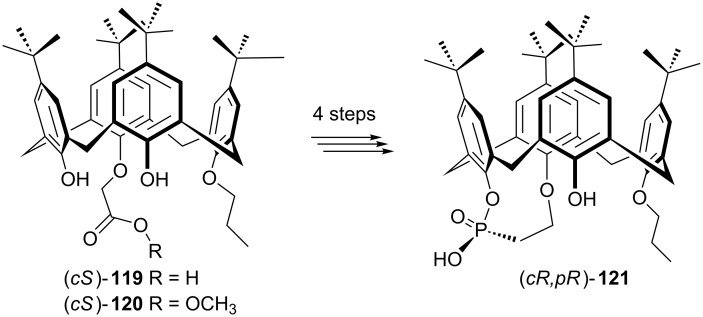
Synthesis of enantiomerically pure inherently chiral calix[4]arene phosphonic acid.

**Scheme 40:**
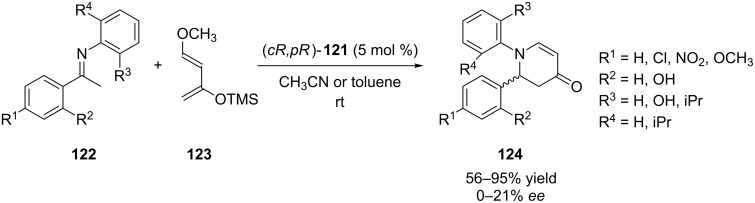
Asymmetric aza-Diels–Alder reactions catalyzed by (*cR,pR*)-**121**.

Manoury et al. have very recently reported facile synthesis of an enantiomerically pure inherently chiral calix[4]arene phosphonic acid (*cR,pR*)-**121** from the readily available (*cS*)-enantiomer of calix[4]arene acetic acid **119** or its methyl ester **120** in four steps (Scheme 39) [73]. The organocatalytic properties of this inherently chiral calixarene Brønsted acid was firstly examined in the aza-Diels–Alder reaction of imines bearing electron-withdrawing or electron-donating substituents **122** with Danishefsky’s diene (**123**, Scheme 40). The corresponding tetrahydropyridine derivatives **124** were obtained in good to excellent yields but the enantioselectivity remained moderate.

**Scheme 41:**
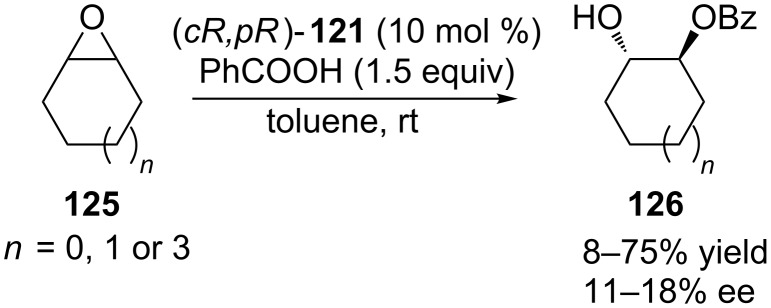
Asymmetric ring opening of epoxides catalyzed by (*cR,pR*)-**121**.

Calixarene phosphonic acid (*cR,pR*)-**121** was also tested in the asymmetric ring opening of several cyclic *meso* epoxides **125** with benzoic acid (Scheme 41). Good yields but low enantiomeric excesses were obtained when cyclohexene and cyclopentene oxides were used as substrates (71% yield, 18% ee; 75% yield, 11% ee, respectively). Cyclooctene oxide was found to be poorly reactive with conversions of less than 10%.

## Conclusion

This review demonstrates that considerable progress in the field of catalytic applications of calixarenes has been achieved in the last ten years. Since the first use of optically active diphosphines based on calix[4]arene skeleton, a remarkable number of new chiral calixarenes have been developed able to effectively catalyze several important transformations: phase-transfer catalysis, Henry reaction, Suzuki–Miyaura cross-coupling and Tsuji–Trost allylic substitution, hydrogenation, Michael addition, aldol and multicomponent Biginelli reactions, epoxidation, Meerwein–Ponndorf–Verley reduction, aza-Diels–Alder and epoxide ring-opening reaction. In these reactions, calixarenes are used either as ligands in metal-catalyzed reactions or as organocatalysts. The application of inherently chiral calixarenes as chiral catalysts is still quite scarce, except in aldol, Michael and Henry reactions. Asymmetric catalysis with chiral calixarenes in which the chirality is induced by attaching chiral groups to the calixarene backbone is more common and the obtained results are more promising. In addition, the calix[4]arene backbone is much more preferred beyond other ring sizes both in transition metal catalysis and organocatalysis. It can be argued that this is due to the difficulty of regiochemical control of calix[6]arene and calix[8]arene functionalization. In calix[4]arene-catalyzed systems, the substrate scope is generally limited to the previously reported ones with small organic molecules. From the summarized studies, although significant progress has been achieved with chiral calix[4]arenes, there are still several opportunities for further enhancements such as catalyst design, loading and substrate scope in the reaction systems. Immobilization of these calixarene derivatives onto polymers or magnetic nanoparticles would also lead to achieve reusable catalysts for batch and continuous-flow studies.

## References

[1] van Leeuwen P W N M (2008). Supramolecular Catalysis.

[2] Karakhanov E E, Maksimov A L, Runova E A, Kardasheva Y S, Terenina M F, Buchneva T S, Guchkova A Ya (2003). Macromol Symp.

[3] Maksimov A L, Sakharov D A, Filippova T Yu, Zhuchkova A Ya, Karakhanov E A (2005). Ind Eng Chem Res.

[4] Maksimov A L, Buchneva T S, Karakhanov E A (2004). J Mol Catal A: Chem.

[5] Gutsche C D (2008). Calixarenes: An Introduction.

[6] Casnati A, Sansone F, Ungaro R, Gokel G W (2003). Calixarene Receptors in Ion Recognition and Sensing. Advances in Supramolecular Chemistry.

[7] Baldini L, Sansone F, Casnati A, Ungaro R, Steed J W, Gale P A (2012). Calixarenes in Molecular Recognition. Supramolecular Chemistry: from Molecules to Nanomaterials.

[8] Hosseinzadeh R, Nemati M, Zadmard R, Mohadjerani M (2016). Beilstein J Org Chem.

[9] Helttunen K, Shahgaldian P (2010). New J Chem.

[10] De Rosa M, La Manna P, Soriente A, Gaeta C, Talotta C, Neri P (2016). RSC Adv.

[11] Simões J B, da Silva D L, de Fátima A, Fernandes S A (2012). Curr Org Chem.

[12] Vita F, Arduini A, Secchi A (2016). Calixarenes and Nanoparticles.

[13] Zhou Y, Li H, Yang Y-W (2015). Chin Chem Lett.

[14] Yaghoubnejad S, Heydar K T, Ahmadi S H, Zadmard R (2018). Biomed Chromatogr.

[15] Mutihac L, Lee J H, Kim J S, Vicens J (2011). Chem Soc Rev.

[16] Homden D M, Redshaw C (2008). Chem Rev.

[17] Bozkurt S, Durmaz M, Naziroglu H N, Yilmaz M, Sirit A (2011). Tetrahedron: Asymmetry.

[18] Durmaz M, Bozkurt S, Naziroglu H N, Yilmaz M, Sirit A (2011). Tetrahedron: Asymmetry.

[19] Durmaz M, Yilmaz M, Sirit A (2011). Org Biomol Chem.

[20] Erdemir S, Yilmaz M (2012). J Inclusion Phenom Macrocyclic Chem.

[21] Sahin O, Memon S, Yilmaz M (2009). J Macromol Sci, Part A: Pure Appl Chem.

[22] Arnott G E (2018). Chem – Eur J.

[23] Kubo Y, Maeda S, Tokita S, Kubo M (1996). Nature.

[24] Maftei C V, Fodor E, Jones P G, Franz M H, Davidescu C M, Neda I (2015). Pure Appl Chem.

[25] Sirit A, Yilmaz M (2009). Turk J Chem.

[26] Szumna A (2010). Chem Soc Rev.

[27] Zheng Y-S, Luo J (2011). J Inclusion Phenom Macrocyclic Chem.

[28] Li Z-Y, Chen J-W, Liu Y, Xia W, Wang L (2011). Curr Org Chem.

[29] Li S-Y, Xu Y-W, Liu J-M, Su C-Y (2011). Int J Mol Sci.

[30] Yilmaz M, Sayin S (2016). Calixarenes in Organo and Biomimetic Catalysis.

[31] Sémeril D, Matt D (2014). Coord Chem Rev.

[32] Shirakawa S, Maruoka K (2013). Angew Chem, Int Ed.

[33] Tan J, Yasuda N (2015). Org Process Res Dev.

[34] Kaneko S, Kumatabara Y, Shirakawa S (2016). Org Biomol Chem.

[35] Srivastava P, Srivastava R (2007). Tetrahedron Lett.

[36] Bozkurt S, Durmaz M, Yilmaz M, Sirit A (2008). Tetrahedron: Asymmetry.

[37] Shirakawa S, Shimizu S (2010). New J Chem.

[38] Huang L, Jin C, Su W (2012). J Chem Res.

[39] Ooi T, Maruoka K (2007). Angew Chem, Int Ed.

[40] De Simone N A, Schettini R, Talotta C, Gaeta C, Izzo I, Della Sala G, Neri P (2017). Eur J Org Chem.

[41] Chang M-L, He Y, Zhou J, Li S-Y (2017). J Braz Chem Soc.

[42] Karpus A, Yesypenko O, Boiko V, Poli R, Daran J-C, Voitenko Z, Kalchenko V, Manoury E (2016). Eur J Org Chem.

[43] Herbert S A, Arnott G E (2009). Org Lett.

[44] Herbert S A, Arnott G E (2010). Org Lett.

[45] Herbert S A, van Laeren L J, Castell D C, Arnott G E (2014). Beilstein J Org Chem.

[46] Liu S, Sandoval C A (2010). J Mol Catal A.

[47] Shirakawa S, Moriyama A, Shimizu S (2008). Eur J Org Chem.

[48] Shirakawa S, Shimizu S (2009). Eur J Org Chem.

[49] Durmaz M, Sirit A (2013). Supramol Chem.

[50] Durmaz M, Sirit A (2013). Tetrahedron: Asymmetry.

[51] Okino T, Hoashi Y, Takemoto Y (2003). J Am Chem Soc.

[52] Durmaz M, Tataroglu A, Yilmaz H, Sirit A (2016). Tetrahedron: Asymmetry.

[53] Genc H N, Sirit A (2016). Tetrahedron: Asymmetry.

[54] Chauhan P, Mahajan S, Kaya U, Hack D, Enders D (2015). Adv Synth Catal.

[55] Zhao B-L, Li J-H, Du D-M (2017). Chem Rec.

[56] Jin C, Zhang M, Deng C, Guan Y, Gong J, Zhu D, Pan Y, Jiang J, Wang L (2013). Tetrahedron Lett.

[57] Gaeta C, Talotta C, Sala P D, Margarucci L, Casapullo A, Neri P (2014). J Org Chem.

[58] Vural U, Durmaz M, Sirit A (2016). Org Chem Front.

[59] Li Z-Y, Lu C-X, Huang G, Ma J-J, Sun H, Wang L, Pan Y (2010). Lett Org Chem.

[60] Eymur S, Akceylan E, Sahin O, Uyanik A, Yilmaz M (2014). Tetrahedron.

[61] Uyanik A, Bayrakci M, Eymur S, Yilmaz M (2014). Tetrahedron.

[62] Angamuthu V, Tai D-F (2015). Appl Catal, A.

[63] Dalpozzo R (2015). Green Chem.

[64] Akceylan E, Uyanik A, Eymur S, Sahin O, Yilmaz M (2015). Appl Catal, A.

[65] Aktas M, Uyanik A, Eymur S, Yilmaz M (2016). Supramol Chem.

[66] Sahin O, Eymur S, Uyanik A, Akceylan E, Yilmaz M (2018). Polycyclic Aromat Compd.

[67] Li Z-Y, Chen Y, Zheng C-Q, Yin Y, Wang L, Sun X-Q (2017). Tetrahedron.

[68] Li Z, Xing H, Huang G, Sun X, Jiang J, Wang L (2011). Sci China: Chem.

[69] Xin J, Chang L, Hou Z, Shang D, Liu X, Feng X (2008). Chem – Eur J.

[70] Bonaccorso C, Brancatelli G, Ballistreri F P, Geremia S, Pappalardo A, Tomaselli G A, Toscano R M, Sciotto D (2014). Dalton Trans.

[71] Nandi P, Matvieiev Y I, Boyko V I, Durkin K A, Kalchenko V I, Katz A (2011). J Catal.

[72] Nandi P, Solovyov A, Okrut A, Katz A (2014). ACS Catal.

[73] Karpus A, Yesypenko O, Boiko V, Daran J-C, Voitenko Z, Kalchenko V, Manoury E (2018). J Org Chem.

